# Assessment of the viability and treatment response of bone metastases in patients with metastatic castration-resistant prostate cancer using choline PET/CT

**DOI:** 10.1097/MD.0000000000026206

**Published:** 2021-06-11

**Authors:** Kazuhiro Kitajima, Shingo Yamamoto, Yusuke Kawanaka, Hisashi Komoto, Kimihiro Shimatani, Takeshi Hanasaki, Motohiro Taguchi, Seiji Nagasawa, Yusuke Yamada, Akihiro Kanematsu, Koichiro Yamakado

**Affiliations:** a Department of Radiology; b Department of Urology, Hyogo College of Medicine, Hyogo, Japan.

**Keywords:** bone metastasis, castration-resistant prostate cancer (CRPC), choline, positioned positron emission tomography computed tomography (PET/CT), treatment response

## Abstract

This study aimed to evaluate the clinical use of choline-PET/CT for discriminating viable progressive osteoblastic bone metastasis from benign osteoblastic change induced by the treatment effect and evaluating the response of bone metastasis to treatment in metastatic castration-resistant prostate cancer (mCRPC) patients. Thirty patients with mCRPC underwent a total of 56 ^11^C-choline-PET/CT scans for restaging, because 4 patients received 1 scan and 26 had 2 scans. Using 2 (pre- and post-treatment) ^11^C-choline-PET/CT examinations per patient, treatment response was assessed according to European Organization for Research and Treatment of Cancer (EORTC) criteria in 20 situations, in which only bony metastases were observed on ^11^C-choline-PET/CT scans. Viable bone metastases and osteoblastic change induced by the treatment effect were identified in 53 (94.6%) and 29 (51.8%) of 56 ^11^C-choline-PET/CT scans, respectively. In 27 cases (48.2%), ^11^C-choline-PET/CT scans could discriminate the 2 entities. The mean SUVmax of the metastatic bony lesions was 5.82 ± 3.21, 5.95 ± 3.96, 6.73 ± 5.04, and 7.91 ± 3.25 for the osteoblastic, osteolytic, mixed, and invisible types, respectively. Of the 20 situations analyzed, CMR, PMR, SMD, and PMD, as determined by the EORTC, were seen in 1, 2, 3, and 14 cases, respectively. Of the 13 patients with increasing PSA trend, all 13 showed PMD. Of the 2 patients with PSA response of <50%, both 2 showed SMD. Of the 5 patients with PSA response of ≥50%, 1 showed CMR, 2 showed PMR, 1 showed SMD, and 1 showed PMD. Choline-PET/CT is very useful to discriminate viable progressive osteoblastic bone metastasis from osteoblastic change, and assess treatment response of bone metastases in mCRPC.

## 1 Introduction

Prostate cancer ranks as the most common lethal malignancy diagnosed and the second leading cause of cancer mortality in western countries. Although high response rates are achieved using androgen blockade as first-line therapy, most men progress toward hormone-refractory prostate cancer. New hormone and systemic chemotherapies have been shown to improve clinical outcome in patients with hormone refractory-prostate cancer; however, they are not curative.^[1,2]^ Due to the high incidence of bone involvement in castration-resistant prostate cancer (CRPC), assessment of treatment response in metastatic prostate cancer to the bone remains a major clinical need, because bone metastases may cause pain, neuropathy, pathologic fracture, and hypercalcemia, which negatively affect patients’ quality of life.^[3]^

Bone metastases in prostate carcinoma are osteoblastic in about 80%, mixed osteoblastic and osteolytic in 15%, and pure osteolytic lesions occasionally (5%).^[4]^ Most advanced prostate cancer patients may harbor both viable osteoblastic bone metastases and treatment-induced sclerosis during the treatment course. Although computed tomography (CT) and bone scintigraphy are widely used to survey these calcified lesions depending on the osteoblastic response induced by tumor cells infiltrating the bone, they have significant limitations in discriminating between these 2 entities.^[5]^ The Response Evaluation Criteria in Solid Tumors (RECIST) regard bone scintigraphy as insufficient appropriate for an evaluation of tumor activity of bone metastases and bone disease is judged as “non-measurable” by RECIST.^[6]^ On the other hand, European Organization for Research and Treatment of cancer imaging group has positioned positron emission tomography computed tomography (PET/CT) using ^11^C-choline or ^18^F-fluorocholine as a potential first choice for monitoring the response of bone metastases to treatment in patients with prostate cancer.^[5]^ Choline PET/CT can directly detect viable bone metastases in prostate cancer, regardless of whether sclerosis is present or not, therefore can discriminate viable progressive osteoblastic bone metastasis from benign osteoblastic change induced by the treatment effect. However, almost no studies have focused the use of choline PET/CT for discriminating viable progressive osteoblastic bone metastasis from benign osteoblastic change induced by the treatment effect and evaluating the response of bone metastasis to treatment in metastatic castration-resistant prostate cancer (mCRPC) patients. Therefore, the usefulness of choline PET/CT for evaluating treatment responses in such cases has yet to be clarified.

The aim of our study is to investigate the feasibility of using choline PET/CT as an imaging biomarker for detecting viable bone metastases directly, discriminating viable progressive osteoblastic bone metastasis from benign osteoblastic change induced by the treatment effect and assessing the treatment response in patients with mCRPC.

## 2 Materials and methods

### 2.1 Patients

The institutional review board approved this study. Informed consent was obtained from each patient after the procedures were fully explained. From October 2015 to September 2019, 30 patients with mCRPC underwent a total of 56 ^11^C-choline PET/CT scans for restaging. The median age and free serum prostate-specific antigen (PSA) level at ^11^C-choline PET/CT scans was 68 years (range 43–84 years) and 13.3 ng/mL (range 0.006–3096 ng/mL). At the time of ^11^C-choline PET/CT scans, 24 (42.9%), 22 (39.3%), 26 (46.4%), and 11 (19.6%) patients had treatment history including new hormonal drugs (abiraterone and enzalutamide), taxane-based chemotherapy (docetaxel and cabazitaxel), radiotherapy, and radium-223 therapy, respectively. Further details of patient demographics are listed in Table 1.

**Table 1:** Patient and tumor characteristics.

	Number or median (range)
Number of choline PET/CT examinations	56
Age at choline PET/CT, y	68 (43–84)
PSA at choline PET/CT, ng/mL	13.3 (0.005–3096)
Initial T stage	
T2/T3/T4	14/28/14
Initial N stage	
Nx/N0/N1/N2	14/25/22/5
Initial M stage	
M0/M1	6/50
Initial PSA, ng/mL	207 (1.55–7013)
Initial Gleason score	
7/8/9/10	3/4/28/21
Previous treatment	
Hormonal treatment	13
Hormonal treatment + Enzalutamide	1
Hormonal treatment + RT	10
Hormonal treatment + RT + Abiraterone	3
Hormonal treatment + RT + Enzalutamide	2
Hormonal treatment + RT + Abiraterone + Enzalutamide	2
Hormonal treatment + RT + Abiraterone + Docetaxel	2
Hormonal treatment + Docetaxel	5
Hormonal treatment + Abiraterone + Docetaxel	6
Hormonal treatment + Abiraterone + Docetaxel + Cabazitaxel	1
Hormonal treatment + Radium-223	3
Hormonal treatment + Radium-223 + Docetaxel	1
Hormonal treatment + RT + Abiraterone + Docetaxel + Radium-223	2
Hormonal treatment + RT + Abiraterone + Docetaxel + Radium-223 + Cabazitaxel	5
Applied therapies between 2 choline PET/CT examinations	
Total	26
Hormonal treatment	1
Abiraterone	6
Enzalutamide	2
Hormonal treatment + RT	2
RT + Abiraterone + Enzalutamide	1
Docetaxel	4
Cabazitaxel	6
Hormonal treatment + Radium-223	4

PET/CT = positron emission tomography/computed tomography, PSA = prostate specific antigen, RT = radiation therapy.

### 2.2 ^11^C-choline PET/CT study

All ^11^C-choline PET/CT examinations were performed using a PET/CT scanner (Gemini TF64; Philips Medical Systems, Eindhoven, the Netherlands). Patients received an intravenous injection of 3.0 MBq/kg body weight ^11^C-choline. The PET/CT scan began 5 minutes after the injection, and emission data were acquired from 7 to 8 bed positions proceeding from the proximal thighs to the base of the skull. Each position required 2 minutes and each scan was acquired in a 3-dimensional (3D) mode. PET images were corrected for random scatter and attenuation, and were reconstructed on a 144-image matrix using an ordered-subsets expectation maximization algorithm (3 iterations, 33 subsets). For attenuation correction and anatomic localization, helical CT scans from the top of the head to the mid-thigh were obtained using the following parameters: tube voltage, 120 kV; effective tube current auto-mA, up to 100 mA; gantry rotation speed, 0.5 seconds; detector configuration, 64 × 0.625 mm; slice thickness, 2 mm; and transverse field of view, 600 mm.

### 2.3 Image analysis

Two experienced nuclear medicine physicians (both readers with 6 years of experience in ^11^C-choline PET/CT), who had no knowledge of the other imaging results or the clinical data, interpreted in consensus the ^11^C-choline PET/CT and intraosseous lesions ≥1.0 cm in the long axis showing abnormal ^11^C-choline uptake were considered active bone metastases. Each bone lesion was classified as the osteoblastic (osteoblastic pattern with bone formation and ossification), osteolytic (osteoclastic pattern with bone resorption), mixed (mixed osteoblastic and osteolytic pattern) or invisible type based on CT findings. Semiquantitative analysis of the abnormal radiotracer uptake for each suspicious bony metastatic lesion was performed using the maximum standardized uptake value (SUVmax). The SUV was calculated as:

SUV=volume of interest (VOI) radioactivity concentration (Bq/mL)/[injected dose (Bq)/patient's weight (g)].

The SUVmax, which was defined as the highest SUV in the pixel with the highest count, within the VOI, was measured and recorded for the focal areas of uptake.

### 2.4 Gold standard

We defined “viable progressive bone metastases” in cases (1) a ^11^C-choline avid bone lesions on ^11^C-choline PET/CT; and (2) positive lesions showing normalization or decreasing of ^11^C-choline uptake in a subsequent ^11^C-choline PET/CT scan or PSA decreasing following therapy; or (3) a progression of the disease on ^11^C-choline PET/CT or PSA increasing during follow-up or after therapy. On the other hand, we defined “benign osteoblastic change induced by the treatment effect” when osteoblastic lesion, being not degenerative change nor benign lesion show no ^11^C-choline uptake on ^11^C-choline PET/CT scan.

### 2.5 Tumor response assessment

According to the European Organization for Research and Treatment of Cancer (EORTC),^[7]^ complete metabolic response (CMR) was considered to be complete resolution of ^11^C-choline uptake within the tumor volume to be indistinguishable from surrounding normal tissue. On the other hand, the appearance of new ^11^C-choline uptake in another region in the second PET/CT scan was classified as progressive metabolic disease (PMD). The EORTC recommends using pre-treatment scan findings to define regions of high ^11^C-choline uptake that represent a viable tumor and also to use the same VOI volumes in subsequent scanning examinations positioned as close to original tumor as possible, as well as measurement of maximal tumor VOI count per pixel per second calibrated as MBq/L. No information regarding the number of lesions to measure is provided by the EORTC, thus we chose up to 5 lesions with the highest level of uptake and up to 2 lesions per organ, and then measured the same lesions in subsequent follow-up scan images.^[8]^ All 5 targets used for SUVmax measurement were summed for each scan, which gave ΣSUVmax. The percentage changes in baseline and second summed SUVmax were calculated. Partial metabolic response (PMR) was defined as a 25% or greater reduction in summed SUVmax value. An increase in the tumor summed SUVmax value of 25% or more within the VOI defined with the baseline scan was classified as PMD, while an increase in the summed SUVmax value of less than 25% or a decrease less than 25% was classified as stable metabolic disease (SMD).

### 2.6 Statistical analysis

We also examined whether there was a significant difference in mean SUVmax among the osteoblastic, osteolytic, mixed, and invisible types using single-factor analysis of variance and a multiple comparison test for parametric data with a Bonferroni correction. A *P* value of less than .05 was considered significant for all analyses. Statistical analyses were performed using SAS software (version 9.3; SAS Institute, Cary, NC, USA).

## 3 Results

**Figure 1:**
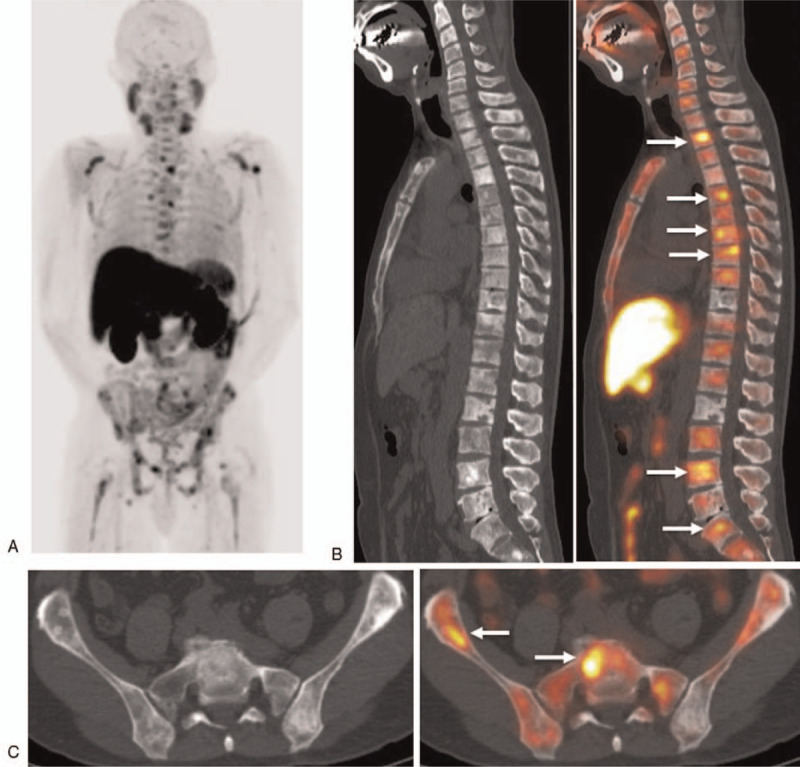
Forty-seven-year-old man with PSA level of 13.41 ng/mL who underwent hormonal therapy and docetaxel for prostate cancer (cT4N1M1b, initial PSA 180 ng/mL, Gleason score 5 + 5), in whom choline PET/CT could discriminate viable progressive osteoblastic bone metastasis from benign osteoblastic change induced by the treatment effect. (A) Maximum intensity projection (MIP) from ^11^C-choline PET/CT image shows abnormal ^11^C-choline uptakes in the spine, pelvic bone, scapula, and femur. (B) Sagittal ^11^C-choline PET/CT image shows multiple osteoblastic lesions in the whole spine with no ^11^C-choline uptakes, reflecting treatment-induced sclerosis during the treatment course and several abnormal ^11^C-choline uptakes in the thoracic (Th2,5,7,8) lumbar spine (L5), and sacral (S1) spine sclerosis (arrows), suggesting viable tumors. (C) Axial ^11^C-choline PET/CT shows several osteoblastic lesions in the sacral spine (S1) and ilium with no ^11^C-choline uptakes, reflecting treatment-induced sclerosis during the treatment course and several abnormal ^11^C-choline uptakes in the right ilium and S1 sclerosis (arrows), suggesting viable tumors.

**Figure 2:**
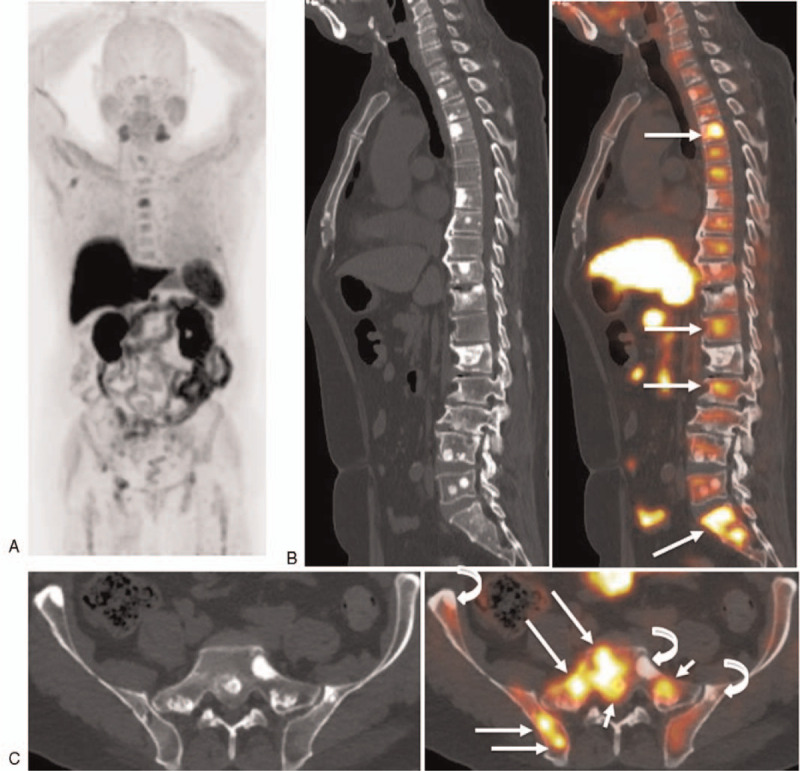
Seventy-five-year-old man with PSA level of 13.41 ng/mL who underwent hormonal therapy and docetaxel for prostate cancer (cT3N0M1b, initial PSA 81.2 ng/mL, Gleason score 5 + 5), in whom choline PET/CT could clearly detect viable progressive osteoblastic bone metastasis, benign osteoblastic change induced by the treatment effect and viable non-osteoblastic bone metastasis. (A) MIP from ^11^C-choline PET/CT image shows abnormal ^11^C-choline uptakes in the spine and pelvic bone. (B) Sagittal ^11^C-choline PET/CT image shows multiple osteoblastic lesions in the spine with no ^11^C-choline uptakes, reflecting treatment-induced sclerosis during the treatment course and several abnormal ^11^C-choline uptakes in the thoracic (Th4,12) lumbar spine (L2), and sacral (S1) spine non-sclerosis (long arrows), suggesting non-osteoblastic bone metastasis. (C) Axial ^11^C-choline PET/CT shows 2 osteoblastic lesions in the sacral spine (S1) and left ilium with no ^11^C-choline uptakes (curved arrow), reflecting treatment-induced sclerosis during the treatment course, 2 abnormal ^11^C-choline uptakes in the S1 sclerosis (short arrows), suggesting viable tumors, and abnormal ^11^C-choline uptakes in the non-osteoblastic lesions of the S1 and right ilium (long arrows), reflecting viable non-osteoblastic bone metastasis.

Viable bone metastases were identified in 53 (94.6%) of 56 ^11^C-choline PET/CT scans (number of bone metastasis = 0, 1–2, 3–10, and ≥11: n = 3 [5.4%], 21 [37.5%], 8 [14.3%], and 24 [42.9%], respectively). On the other hand, benign osteoblastic change induced by the treatment effect were identified in 29 (51.8%) of 56 ^11^C-choline PET/CT scans (number of benign osteoblastic change = 0, 1–5, 6–10, and ≥11: n = 27 [48.2%], 9 [16.1%], 8 [14.3%], and 12 [21.4%], respectively). Among these 29 ^11^C-choline PET/CT scans showing benign osteoblastic change induced by the treatment effect, 27 ^11^C-choline PET/CT scans proved viable bone metastases, in which ^11^C-choline PET/CT could discriminate viable progressive osteoblastic bone metastasis from benign osteoblastic change induced by the treatment effect. Two representative cases are shown in Figures 1 and 2.

Among all 615 metastatic bony lesions, 292 (47.5%) were osteoblastic, 25 (4.1%) were osteolytic, 16 (2.6%) were mixed, and 282 (45.9%) were invisible according to the CT morphological types. The mean SUVmax of all 615 metastatic bony lesions was 6.79 ± 3.46 (range 1.78–20.01). The mean SUVmax of the metastatic bony lesions was 5.82 ± 3.21 (range 1.78–20.01), 5.95 ± 3.96 (range 1.8–17.17), 6.73 ± 5.04 (range 2.14–15.15), and 7.91 ± 3.25 (range 2.55–16.33) for the osteoblastic, osteolytic, mixed, and invisible types, respectively. SUVmax differed significantly among the 4 subgroups (*P* < .0001). Moreover, a Bonferroni correction revealed significant differences in SUVmax between the osteoblastic and invisible types (*P* = .016), and between the osteolytic and invisible types (*P* = .035). No significant difference in SUVmax was observed between the osteoblastic and osteolytic types.

Intra-prostate local recurrence, lymph node metastasis, and visceral metastases were identified in 9 (16.1%), 13 (23.2%), and 7 (12.5%) ^11^C-choline PET/CT scans, respectively.

### 3.1 Response assessment

Using 2 (pre- and post-treatment) ^11^C-choline PET/CT examinations per patient, treatment response was assessed in 26 situations. Applied therapies in 26 situations were hormonal therapy (n = 1), new hormonal drugs (n = 8), hormonal therapy and radiotherapy (n = 2), radiotherapy and new hormonal drugs (n = 1), taxane-based drugs (n = 10), and hormonal therapy and radium-223 therapy (n = 4). Excluding 6 situations in which intra-prostate, nodal, or visceral metastases were observed on ^11^C-choline PET/CT scans, 20 situations with only bony metastases on ^11^C-choline PET/CT scans were used to assess the treatment response. Of the 20 situations analyzed, CMR, PMR, SMD, and PMD, as determined by the EORTC criteria, were seen in 1 (5.0%), 2 (10.0%), 3 (15.0%), and 14 (60.0%) cases, respectively. An increasing PSA trend was seen in 13 patients (65.0%) and a decreasing PSA trend in 7 patients (35.0%). A PSA response of ≥50% was seen in 5 patients (25.0%). Of the 13 patients with an increasing PSA trend, all 13 (100%) showed PMD. Of the 2 patients with a PSA response of <50%, both 2 (100%) showed SMD. Of the 5 patients with a PSA response of ≥50%, 1 (20.0%) showed CMR, 2 (40.0%) showed PMR, 1 (20%) showed SMD, and 1 (20.0%) showed PMD (Table 2).

**Table 2:** Comparison between EORTC criteria and PSA response to treatment.

	PSA response		
	Decrease ≥50%	Decrease <50%	Increasing PSA trend
CMR	1	0	0
PMR	2	0	0
SMD	1	2	0
PMD	1	0	13
Total	5	2	13

CMR = complete metabolic response, EORTC = European Organization for Research and Treatment of Cancer, PMD = progressive metabolic disease, PMR = partial metabolic response, PSA = prostate specific antigen, SMD = stable metabolic disease.

## 4 Discussion

This is the first study to focus the clinical utility of choline PET/CT for detecting viable bone metastases directly, discriminating viable progressive osteoblastic bone metastasis from benign osteoblastic change induced by the treatment effect and evaluating the response of bone metastasis to treatment in mCRPC patients. In our series, we clarified that (1) ^11^C-choline PET/CT could directly detect viable bone metastases with abnormal ^11^C-choline uptake, regardless of various morphological types (osteoblastic, osteolytic, mixed, and invisible types on CT), (2) ^11^C-choline PET/CT could discriminate viable progressive osteoblastic bone metastasis (abnormal ^11^C-choline uptake) from benign osteoblastic change induced by the treatment effect (no ^11^C-choline uptake) on ^11^C-choline PET/CT, (3) treatment response (EORTC criteria) corresponded well with changes in the serum PSA level.

Beheshti et al reported that bone metastasis of advanced prostate cancer could be categorized into 3 groups based on the findings of ^18^F-fluorocholine PET/CT as follows: (a) bone marrow involvement (positive on choline PET and negative on CT); (b) typically osteoblastic but less often osteoclastic lesions (positive on choline PET and CT); and (c) densely sclerotic lesions (negative on choline PET and positive on CT). The densely sclerotic lesions that exhibited no metabolic uptake of choline could be attributable to the therapy-induced apoptosis of cancerous cells.^[9]^

Several studies have evaluated the use of choline PET/CT for the assessment of treatment response in patients with mCRPC.^[10–12]^ Ceci et al investigated the role of ^11^C-choline PET/CT in evaluating the response to docetaxel in 61 cases with mCRPC and compared the metabolic response evaluated using ^11^C-choline PET/CT to the PSA response.^[10]^ Of the 29 patients with an increasing PSA trend, all 23 showed PMD and 6 showed SMD. Of the 32 patients with a decreasing PSA trend, 16 showed PMD, 8 showed SMD, 2 showed PMR, and 6 showed CMR. Of the 25 patients with a PSA response of ≥50%, 11 showed PMD, 6 showed SMD, 2 showed PMR, 6 showed CMR. De Giorgi et al assessed the usefulness of ^18^F-choline PET/CT for evaluating mCRPC with regard to their early response to treatment with abiraterone (n = 43) or enzalutamide (n = 36).^[11,12]^ The authors concluded that both metabolic response, assessed using ^18^F-choline PET/ CT findings and PSA response greater than or equal to 50% alone were both associated with more favorable progression-free survival and overall survival.

In recent years, whole-body diffusion weighted magnetic resonance imaging^[13,14]^ and ^68^Ga prostate-specific membrane antigen (^68^Ga-PSMA) PET/CT^[15,16]^ have emerged as a new imaging modality for monitoring the response of bone metastases to treatment in patients with prostate cancer. Yoshida et al demonstrated that the extent of bone metastasis and the presence of visceral metastasis on whole-body diffusion weighted imaging (WB-DWI) using METastasis Reporting and Data System for Prostate Cancer (MET-RADS-P) score were significantly associated with a shorter cancer specific survival in 72 mCRPC patients.^[14]^ Grubmüller et al demonstrated that PSMA-PET parameters’ change (SUV and PSMA total tumor volume) were significantly associated with PSA response to systemic therapies for mCRPC in 43 patients.^[16]^

This study had several limitations. First, the sample size of this single institution is too small to draw a definite conclusion. Therefore, a prospective, multicenter trial including a large cohort of patients would help better clarify the exact role of ^11^C-choline PET/CT in clinical decision-making and long-term outcomes in this clinical setting. Second, the population of the enrolled patients was heterogenous; treatment procedure patients. This sample heterogeneity will bring complicated confounding factors for analysis. Third, the gold standard for any analysis is the histological confirmation of the findings. However, clinical follow-up is a valid approach for evaluation of diagnostic accuracy and response to therapy, and it would have been unethical to investigate all PET/CT-detected bony lesions using invasive procedures. Positive findings are easy to confirm, but negative findings only indicate that it has not been possible to acquire positive findings during follow-up, making it uncertain whether the findings are truly negative. We have not experienced some benign lesion which present as high choline uptake and normal finding in CT image in 30 patients. Forth, new and more sensitive PET tracers for prostate cancer, such as ^18^F-FACBC^[17]^ and ^68^Ga-PSMA,^[18]^ were recently introduced for clinical use in western countries, but they are not yet available in Japan. ^18^F-sodium fluoride (NaF) PET/CT is a highly sensitive method for evaluating osteoblastic bone metastasis and used for treatment monitoring in patients with progressive osseous metastasis, however NaF PET/CT is not tumor specific and is subject to the flare phenomenon associated with systemic therapy.^[19]^ Fifth, the inter- and intra-observer agreement on the ^11^C-choline PET/CT have not been evaluated, because that is not the principal objective of this study.

In conclusion, choline-PET/CT is very useful to directly determine the viability of bone metastases, regardless of whether sclerosis is present or not, and discriminate viable progressive osteoblastic bone metastasis from benign osteoblastic change induced by the treatment effect. Moreover, choline-PET/CT could assess treatment response of bone metastases and enable us to design optimal treatment strategies for patients with mCRPC.

## Author contributions

**Akihiro Kanematsu:** accrural of patients, reviewing manuscript

**Hisashi Komoto:** study design, reviewing manuscript

**Kazuhiro Kitajima:** writing manuscript, statistical analysis, reviewing manuscript

**Kimihiro Shimatani:** accrural of patients, reviewing manuscript

**Koichiro Yamakado:** study design, reviewing manuscript, overall supervise

**Motohiro Taguchi:** accrural of patients, reviewing manuscript

**Seiji Nagasawa:** accrural of patients, reviewing manuscript

**Shingo Yamamoto:** study design, accrural of patients, reviewing manuscript

**Takeshi Hanasaki:** accrural of patients, reviewing manuscript

**Yusuke Kawanaka:** study design, reviewing manuscript

**Yusuke Yamada:** accrural of patients, reviewing manuscript

**Conceptualization:** Kazuhiro Kitajima, Shingo Yamamoto.

**Data curation:** Kazuhiro Kitajima, Shingo Yamamoto, Yusuke Kawanaka, Hisashi Komoto, Kimihiro Shimatani, Takeshi Hanasaki, Motohiro Taguchi, Seiji Nagasawa, Yusuke Yamada, Akihiro Kanematsu, Koichiro Yamakado.

**Formal analysis:** Kazuhiro Kitajima.

**Investigation:** Kazuhiro Kitajima.

**Methodology:** Kazuhiro Kitajima.

**Project administration:** Kazuhiro Kitajima.

**Resources:** Kazuhiro Kitajima.

**Software:** Kazuhiro Kitajima.

**Supervision:** Koichiro Yamakado.

**Writing – original draft:** Kazuhiro Kitajima.

**Writing – review & editing:** Kazuhiro Kitajima, Shingo Yamamoto, Yusuke Kawanaka, Hisashi Komoto, Kimihiro Shimatani, Takeshi Hanasaki, Motohiro Taguchi, Seiji Nagasawa, Yusuke Yamada, Akihiro Kanematsu, Koichiro Yamakado.
